# Exosomes Secreted by Adipose-Derived Mesenchymal Stem Cells Foster Metastasis and Osteosarcoma Proliferation by Increasing COLGALT2 Expression

**DOI:** 10.3389/fcell.2020.00353

**Published:** 2020-05-25

**Authors:** Yan Wang, Yijing Chu, Kun Li, Guoqing Zhang, Zhu Guo, Xiaolin Wu, Chensheng Qiu, Yan Li, Xin Wan, Jing Sui, Dan Zhang, Hongfei Xiang, Bohua Chen

**Affiliations:** ^1^Department of Spinal Surgery, Qingdao University Affiliated Hospital, Qingdao, China; ^2^Department of Obstetrics and Gynecology, The Affiliated Hospital of Qingdao University, Qingdao, China; ^3^Department of Hepatobiliary and Pancreatic Surgery, The Affiliated Hospital of Qingdao University, Qingdao, China; ^4^Department of Medicine, Qingdao University, Qingdao, China

**Keywords:** exosomes, adipose-derived mesenchymal stem cells, osteosarcoma, Homo sapiens collagen beta (1-O)galactosyltransferase 2, metastasis

## Abstract

**Objectives:**

Homosapien collagen beta (1-O) galactosyl transferase 2 (COLGALT2) is an important enzyme during collagen glycosylation, yet its biological functions in cancer are incompletely understood. Our previous study revealed that in the osteosarcoma microenvironment, adipose-derived mesenchymal stem cells (ADSCs) demonstrate cancer-promoting effects, but the exact mechanisms remain unclear. The aim of this study was to investigate the role of COLGALT2 in the osteosarcoma-fostering effects of ADSCs.

**Materials and Methods:**

In this study, we compared COLGALT2 expression between primary and metastatic osteosarcoma tissues and found that metastatic tissues expressed significantly higher COLGALT2 levels. Then, we isolated and identified exosomes secreted by ADSCs. Additionally, we assessed the roles of ADSC exosomes and COLGALT2 in the osteosarcoma-promoting effects of ADSCs.

**Results:**

Our results showed that ADSC exosomes could foster the invasion, migration, and proliferation of osteosarcoma cells, together with increasing COLGALT2 expression. COLGALT2 inhibition in MG63 cells suppressed the ADSC exosome-mediated fostering of osteosarcoma cell invasion, migration and proliferation *in vitro*. Conversely, COLGALT2 overexpression promoted U-2OS cell invasion, migration and proliferation *in vitro*. Additionally, COLGALT2 inhibition attenuated metastasis and tumor growth, and ADSC exosomes promoted tumor progression, as demonstrated in a nude mouse model of osteosarcoma.

**Conclusion:**

According to these data, ADSC exosomes foster osteosarcoma progression by increasing COLGALT2 expression in osteosarcoma cells.

## Introduction

In children and adolescents, osteosarcoma is the most common and devastating bone cancer. Moreover, the 5-year survival rate is no more than 70% because of tumor recurrence caused by metastasis and drug resistance (Anderson, 2016). The ever-increasing evidence indicates a network between cancer cells and the tumor microenvironment that fuels disease progression and metastasis (Avril et al., 2016; Baumann and Hennet, 2016). Osteosarcoma recurrence has been revealed after the use of autologous fat grafts, and adipose-derived stem cells (ADSCs) are abundant in intermuscular tissue. Further observation has shown that ADSCs have a tumor-promoting effect in osteosarcoma (Bonuccelli et al., 2014; Chen et al., 2018). Our previous work revealed that ADSCs stimulate osteosarcoma metastasis and proliferation through sensitizing STAT3 signaling (Collantes et al., 2018). The exact mechanisms underlying these effects remain poorly understood, although much evidence clearly indicates that ADSCs in the microenvironment contribute to tumor progression. Exosomes are extracellular vesicles that transport lipids, messenger RNA, DNA, microRNA, and proteins and are crucial players in intercellular communication (De Oliveira et al., 2010). Exosomes derived from stromal cells are integral for formation of the tumor microenvironment and have been involved in all stages of cancer progression (Guiho et al., 2018). Understanding the effects of ADSC exosomes in the osteosarcoma microenvironment may allow ADSCs to serve as cancer prevention targets for cancer therapy.

The recently identified COLGALT2 gene encodes Hyl-specific galactosyltransferase enzymes, which initiate collagen glycosylation in the endoplasmic reticulum (Guo et al., 2019; Hennet, 2019). In the organizational barriers of the human body, collagens are the richest proteins, and collagen post-translational modifications are important in diseases related to matrix remodeling; therefore, these molecules are likely to modulate the spreading and adhesion of cells on basement membranes (Lastra and Manrique, 2015; Hennet, 2019). The inactivation of COLGALT1 in osteosarcoma cells led to collagen type I accumulation, and cells with inactive COLGALT1 and COLGALT2 genes could not be grown, suggesting that osteosarcoma cell viability and proliferation are impaired by a complete loss of collagen glycosylation (Lee et al., 2015). However, at present, we have no knowledge of the role of COLGALT2 in the osteosarcoma-promoting effects of ADSCs.

In our research, we identified and isolated exosomes from ADSCs to assess the influence of COLGALT2 when ADSC osteosarcoma and exosomes were interacting. We predicted that ADSC exosomes could increase COLGALT2 expression in osteosarcoma, which would promote osteosarcoma proliferation and osteosarcoma cell invasion. We examined the potential roles of COLGALT2 in osteosarcoma and ADSC exosomes in communication between ADSCs and osteosarcoma cells.

## Materials and Methods

### Exosome Isolation

Exosomes were obtained from ADSC supernatants by differential centrifugation. The medium was discarded when ADSCs reached 70% confluence. Then, the cells were cultured in serum-free DMEM/F12 for another 24 h. The supernatants were collected and then cleared by sequential centrifugation at 15,000 × g for 30 min or 3,000 × g for 30 min. The supernatants were ultracentrifuged at 120,000 × g for 2 h after filtration with 0.22-mm filters (Millipore, Billerica, MA). The exosomes were washed and collected several times with sterile PBS. The exosome concentrations were measured with a Pierce BCA protein assay kit (Thermo Fisher Scientific).

### Electron Microscopy

For ∼10 min, almost 50 μl of prepared exosomes was adsorbed and placed onto formvar carbon-coated 300-mesh copper grids. Then, the adsorbed exosomes were dried at room temperature for 30 min and negatively dyed with 3% phosphotungstic acid. Finally, the exosomes were studied using a transmission electron microscope (Olympus Software Imaging Solutions) at 120.0 kV. Moreover, a digital camera was used to capture the exosomes.

### Immunohistochemistry

Tumor tissues were split into 4-μm sections and embedded in paraffin. After the tissues were dehydrated in a graded alcohol series, antigen retrieval was conducted at 4°C with 100 μl of a solution that contained antibodies against Ki67 and COLGALT2 (1:200 dilution, Abcam MA, United States). Moreover, the sections were incubated for 20 min at 37°C to develop the signal from a diluted biotinylated secondary antibody. To visualize the target proteins, fresh 3,3-diaminobenzidine (DAB) solution was applied, and hematoxylin was performed as a tissue counterstain. Two observers independently assessed the expression of target proteins with an Olympus FV500 microscope (Olympus, Tokyo, Japan). Image-Pro Plus 5.1 was used to evaluate the protein expression levels and investigate the intensity and area of staining in five random regions (× 200 magnification).

### Immunofluorescence

With or without purified ADSC exosomes (10 μg/ml), osteosarcoma cells were developed for 24 h. With 4% paraformaldehyde for 60 min, all of the cells were then gathered, separated and settled. The settled cells were cut into 4-μm sections and placed in paraffin. Then, the sections were washed three times with PBS, blocked with 10% goat serum for 1 h and subsequently washed two times with 0.2% Triton X-100. Next, the cells were incubated with primary antibodies (COLGALT2 and vimentin, purchased from Abcam, MA, United States), secondary antibodies (Invitrogen) and DAPI (Guangzhou RiboBio, Guangzhou, China). Images were captured using a fluorescence microscope.

### Proliferation and Invasion Assays

Osteosarcoma cells were incubated with purified exosomes (10 μg/ml) for 48 h and used for the subsequent experiments. Cell invasion and growth assays were conducted according to the manufacturer’s instructions as described in previous studies (Collantes et al., 2018).

### Colony Formation Assay

In total, 500–1,000 osteosarcoma cells were cultured in six-well plates for 14 days for the colony formation assay. Next, the colonies were dyed with a crystal violet solution for visualization and fixed with paraformaldehyde.

### Wound-Healing Assay

Osteosarcoma cells were cultured in a 6-well plate at 4 × 10^5^ cells per well. A 200-μl pipette tip was used to scratch a straight line in each well after the cells had attached completely. The cells were incubated in serum-free medium for an additional 24 h and washed with PBS. The scratch lines were monitored with a camera attached to a microscope.

### shRNA Transfection

Osteosarcoma cells were placed in 10-cm plates at a density of 5 × 10^5^ cells per well and incubated for 24 h. Then, they were transfected with a scrambled shRNA control (shNC) (Genechem, Shanghai, China) or COLGALT2-specific shRNA using Lipofectamine 2000 (Invitrogen, Carlsbad, CA, United States).

### qRT-PCR Assay and RNA Isolation

Osteosarcoma cells were collected. Additionally, RNA was isolated using TRIzol reagent (Invitrogen, Carlsbad, United States). Then, 2 μg of RNA was used for first-strand cDNA synthesis, which was performed using a reverse transcription kit (Toyobo, Osaka, Japan). Quantitative real-time (qRT) PCR was conducted with master mix (Thermo Fisher Scientific) and gene-specific TaqMan probes (Applied Biosystems) according to the manufacturer’s instructions. GAPDH expression was examined in osteosarcoma cells treated with ADSC exosomes or Colgalt2 shRNA to confirm the best housekeeping gene for RNA normalization in qRT-PCR experiments (Supplementary Figure S1). Our results showed that the Ct value of GAPDH mRNA was high and stable in both MG63 and U2-OS cells; therefore, the qRT-PCR results were normalized to GAPDH expression. The TaqMan probes used included COLGALT2, Hs00967230_m1; GAPDH, Hs02786624_g1; vimentin, Hs00418522_m1; MMP-9, Hs00957562_m1; and MMP-2, Hs01548727_m1.

### Western Blotting

To remove cell debris, osteosarcoma cells were centrifuged at 10,000 g and lysed for 10 min on ice in NP40 buffer (Beyotime, Shanghai, China). Equal amounts (30 μg) of protein in cell extracts were resolved by SDS-PAGE and transferred to polyvinylidene fluoride (PVDF) membranes (Bio-Rad, Hercules, CA). Then, the transferred membranes were cultured with primary antibodies against beta-actin (1:2,000 dilution; Abcam, MA, United States) and human TSG101, CD63, MMP9 and MMP2 (1:1,000; Cell Signaling Technology). Next, the membranes were incubated with peroxidase-conjugated AffiniPure secondary IgG antibodies (H + L) (1:2,000; R&D Systems). The protein-antibody complexes were quantitated and detected using Image Lab^TM^ version 5.1 software (Bio-Rad, Hercules, CA) in a chemiluminescence detection system.

### *In vivo* Metastasis Assays and Tumor Growth

The Animal Ethics Committee of the Affiliated Hospital of Qingdao University approved all experimental protocols involving animals. Three- to four-week-old female BALB/c nude mice were purchased from the Shanghai Animal Centre (Shanghai, China). Moreover, to construct the xenograft model, MG63 cells (8 × 10^6^ cells/mouse) were injected into the right proximal tibia. After the injection of MG63 cells, COLGALT2 shRNA/shControl (once per week) and ADSC exosomes (20 μg, twice per week) were injected into the tumors for 1 week. Six mice were included in each of the four groups. Each mouse was injected with MG63 cells + shControl + exosomes, MG63 cells + shControl + PBS, MG63 cells + shRNA + exosomes or MG63 cells + shRNA + PBS. After receiving cell injections for 25 days, the animals were sacrificed.

As described above, the luminescence of the MG63 cells in the tibia marrow cavity was measured (Collantes et al., 2018).

### Statistical Analysis

The data in our study are expressed as the means ± standard deviations. We calculated the means after conducting at least three independent experiments. The significance of differences was analyzed with one-way analysis of variance or a two-tailed Student’s *t*-test. A value of *P* < 0.05 was regarded as significant.

## Results

### COLGALT2 Is Upregulated in Metastatic Osteosarcoma Tumors

**FIGURE 1:**
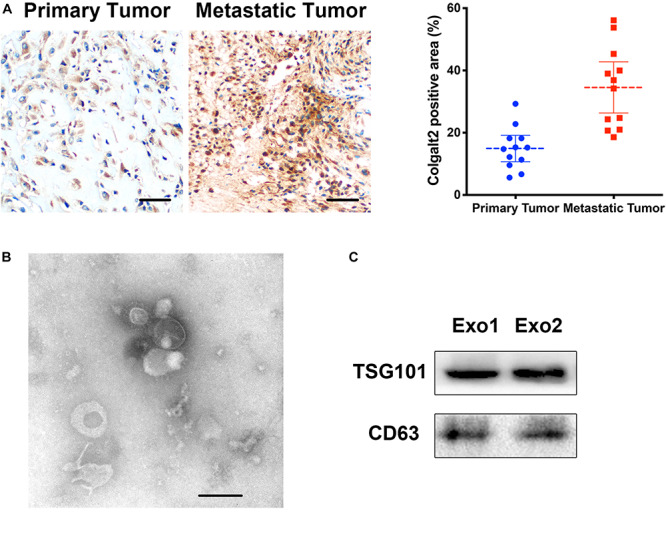
COLGALT2 overexpression in metastatic osteosarcoma tissues and ADSC exosome characterization. **(A)** Immunohistochemical analysis of COLGALT2 protein expression in human primary and metastatic osteosarcoma tissues. **(B)** ADSC exosomes were visualized by electron microscopy (×30,000). Scale bar = 100 nm. **(C)** Western blotting shows that ADSC exosomes expressed exosome-associated proteins, including CD63 and TSG101.

Because the expression of COLGALT2 may be related to tumor metastasis, we examined COLGALT2 expression in patients with osteosarcoma (using 12 paired primary and metastatic tumor tissues; all metastatic tumor tissues were from the lungs). Our results showed that COLGALT2 was significantly upregulated in metastatic osteosarcoma tumors (Figure 1A), which indicated a relationship between COLGALT2 expression and osteosarcoma progression.

### ADSC Exosomes Enhance Osteosarcoma Cell Invasion, Migration, and Proliferation *in vitro*

**FIGURE 2:**
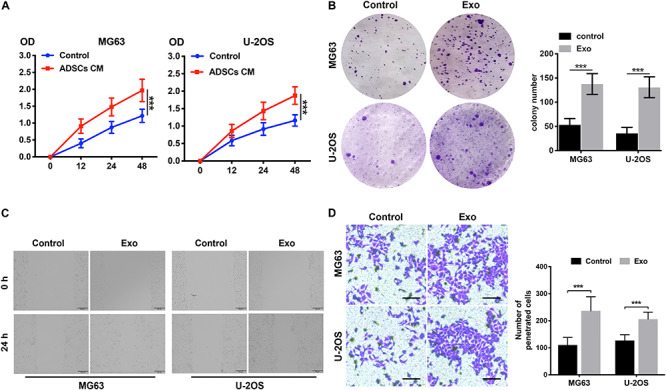
ADSC exosomes foster the invasion, migration, and proliferation of osteosarcoma cells. **(A)** The effects of ADSC exosomes on osteosarcoma cell proliferation were assessed by a CCK8 assay. Osteosarcoma cells were treated with ADSC exosomes, and the optical densities of the two groups were measured at 450 nm. Data collected from the three respective experiments are displayed. **(B)** Statistical analysis of colony numbers and representative images of colony formation are shown. **(C)** Wound-healing assay comparing the motility of osteosarcoma cells treated with or without ADSC exosomes. **(D)** Comparison of osteosarcoma cell invasion after treatment with or without ADSC exosomes using Transwell compartments. The statistical analysis of the number of penetrated cells is shown in the right panel. ***P < 0.001.

Exosomes were purified from ADSC supernatants, and the donor information is listed at Supplementary Table S1. An electronic microscope (EM) was used to measure the diameters of the purified exosomes. All types of exosomes were round vesicles with diameters of ∼50–150 nm (Figure 1B). Then, western blot analysis was used to detect exosome-associated proteins containing CD63 and TSG101 (Figure 1C). In order to determine the effects of exosomes on osteosarcoma cell proliferation, we determined cell viability after adding 10 μg/ml exosomes to two osteosarcoma cell lines for 48 h. We found that treatment with exosomes derived from ADSCs significantly promoted the proliferation of the two osteosarcoma cell lines (Figure 2A). Colony formation assays indicated that the colony numbers and size were increased by exosomes (Figure 2B). Next, we examined whether the exosomes collected from ADSC supernatants could augment osteosarcoma cell invasion and migration. Wound-healing assays showed that ADSC exosomes significantly enhanced the migratory ability of the two osteosarcoma cell lines (Figure 2C). ADSC exosomes significantly increased the invasive capabilities of the osteosarcoma cells, as shown by the Transwell Matrigel invasion assays (Figure 2D). These results revealed that exosomes have a marked influence on the osteosarcoma-promoting effect of ADSCs.

### ADSC Exosomes Induce COLGALT2 Upregulation in Osteosarcoma Cells, Accompanied by Increased Vimentin and MMP2/9 Expression

**FIGURE 3:**
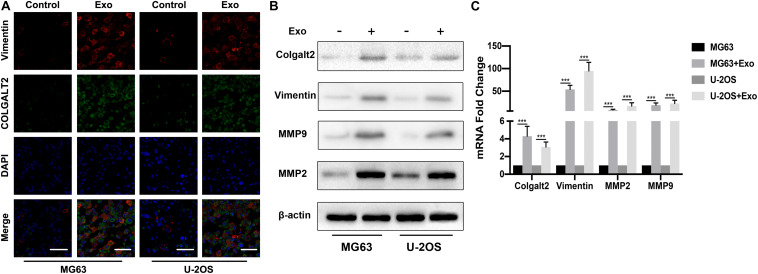
ADSC exosomes induce COLGALT2 upregulation in osteosarcoma cells, accompanied by increased vimentin and MMP2/9 expression. **(A)** Representative IF images of vimentin and COLGALT2 in osteosarcoma cells treated with or without ADSC exosomes. Scale bar = 25 μm. **(B)** Western blotting shows that in osteosarcoma cells, ADSC exosome treatment upregulated the expression of COLGALT2, vimentin, MMP9 and MMP2. **(C)** Real-time PCR displays the relative mRNA expression levels of COLGALT2, vimentin, MMP9 and MMP2 in osteosarcoma cells treated with or without ADSC exosomes. ***P < 0.001.

To understand the role of COLGALT2 in the osteosarcoma-promoting effect of ADSC exosomes, we first examined COLGALT2 and vimentin expression in exosome-treated osteosarcoma cells by immunofluorescence. As controls, osteosarcoma cells were cultured alone. ADSC exosome-treated tumor cells expressed higher levels of COLGALT2 and vimentin than control cells (Figure 3A). Then, in osteosarcoma cells, we evaluated COLGALT2, vimentin and matrix metalloproteinase 2/9 (MMP2/9) expression together with ADSC exosomes using qRT-PCR and western blotting. Exosome treatment markedly increased COLGALT2, vimentin and MMP2/9 expression in the osteosarcoma cells (Figures 3B,C). To determine the possible mechanism that leads the ADSCs exosomes to increase COLGALT2 expression in osteosarcoma cells, we examined the mRNA level of COLGALT2 in ADSCs exosomes compared with MRC-5 cells exosomes, and higher COLGALT2 mRNA level was found in ADSCs exosomes (Supplementary Figure S2).

### Osteosarcoma Cells Overexpress COLGALT2

**FIGURE 4:**
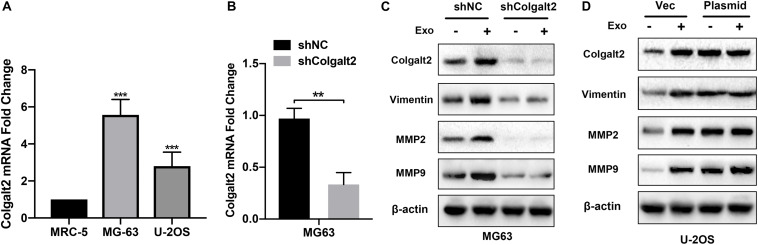
Overexpression of COLGALT2 and confirmation of COLGALT2 inhibition and overexpression in osteosarcoma cells. **(A)** qRT-PCR analysis shows significantly upregulated COLGALT2 mRNA expression in MG63 and U-2OS cells compared to MCR-5 cells. **(B)** qRT-PCR analysis showed significantly decreased COLGALT2 mRNA expression in MG63 cells treated with COLGALT2 shRNA. **(C)** Immunoblotting assays showed that COLGALT2 inhibition decreased the protein expression of COLGALT2, vimentin and MMP2/9 in MG63 cells. **(D)** Immunoblotting assays show that COLGALT2 overexpression increased the protein expression of COLGALT2, vimentin, and MMP2/9 in U2-OS cells. ***P < 0.001, **P < 0.01.

To characterize the role of COLGALT2 in the osteosarcoma-promoting effects of exosomes, we first tested COLGALT2 expression levels by qRT-PCR in two osteosarcoma cell lines. For the control, the normal fibroblast cell line MRC5 was applied. However, the two osteosarcoma cell lines expressed higher levels of COLGALT2 than the MRC5 cells; moreover, compared with that in U-2OS cells, COLGALT2 expression was remarkably greater in MG63 cells (Figure 4A). Then, we inhibited COLGALT2 expression in MG63 cells using shRNA and overexpressed COLGALT2 in U-2OS cells using a plasmid. Next, we confirmed COLGALT2 mRNA expression in MG63 cells (Figure 4B). The COLGALT2 shRNA and overexpression plasmid respectively decreased and increased vimentin and MMP2/9 protein expression in U-2OS cells and MG63 cells (Figures 4C,D).

### ADSC Exosomes Promote Osteosarcoma Cell Invasion, Migration, and Proliferation via COLGALT2

**FIGURE 5:**
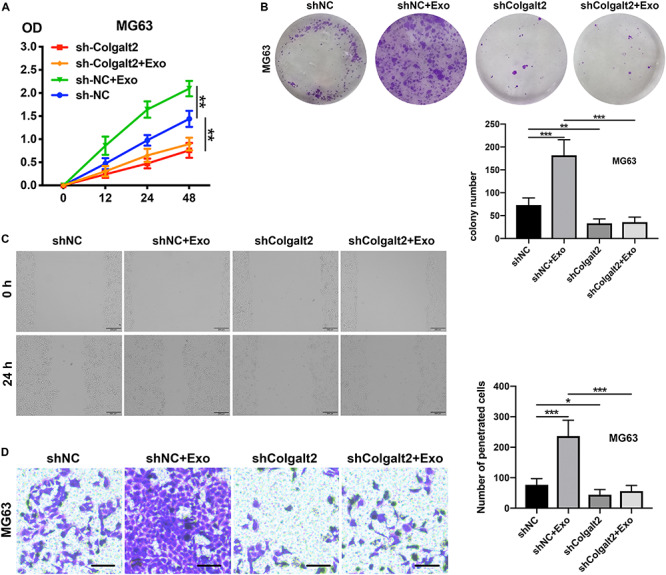
COLGALT2 inhibition attenuates the osteosarcoma-promoting effects of ADSC exosomes. **(A)** The effects of COLGALT2 inhibition on MG63 cell proliferation were assessed with a CCK8 assay. Data collected from the three respective experiments are reflected. **(B)** Representative images of colony numbers (right panel) and colony formation (left panel) after COLGALT2 knockdown in MG63 cells. **(C)** Representative images of wound areas at 24 h after COLGALT2 knockdown in MG63 cells. MG63 cells that were cultured alone served as controls. **(D)** Representative images and statistical analysis of invaded cells in the Transwell invasion assay. All values are presented as the means ± SDs of three independent experiments. ***P < 0.001, **P < 0.01, *P < 0.05.

After confirming the inhibitory efficacy of COLGALT2 shRNA in MG63 cells, our team tested the impact of COLGALT2 inhibition on MG63 cell invasion, migration, and proliferation. Consistent with the decreased expression of vimentin and MMP2/9, we observed that COLGALT2 inhibition attenuated the significant increase in cell invasion, migration and proliferation in MG63cells after ADSC exosome treatment (Figures 5A–D).

**FIGURE 6:**
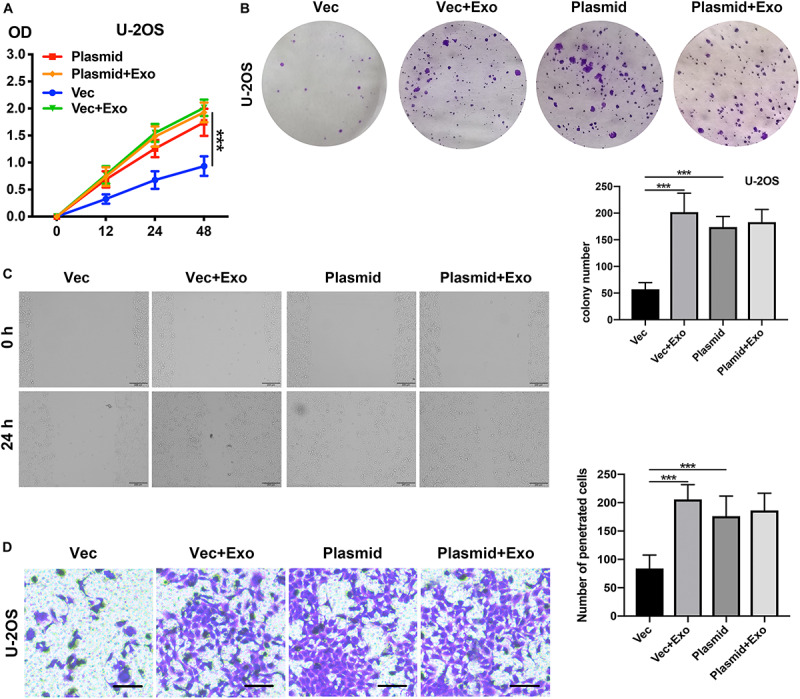
COLGALT2 overexpression increases U-2OS cell invasion, migration, and proliferation. **(A)** The effects of COLGALT2 overexpression on the proliferation of U-2OS cells were evaluated using a CCK8 assay. Data collected from the three respective experiments are displayed. **(B)** Colony numbers (right panel) and representative images of colony formation (left panel) after COLGALT2 overexpression in U-2OS cells. **(C)** After COLGALT2 overexpression in U-2OS cells, representative images of wound areas at 24 h and U-2OS cells cultured alone as controls. **(D)** Representative images and statistical analysis of invaded cells in the Transwell invasion assay. All values are presented as the means ± SDs of three independent experiments. ***P < 0.001.

Given our observation of the relationship between COLGALT2 and the tumor-promoting effects of ADSC exosomes, we next investigated whether COLGALT2 overexpression had tumor-promoting effects similar to those of ADSC exosomes. We used the COLGALT2 overexpression plasmid to increase COLGALT2 expression in U-2OS cells. Similar to ADSC exosome treatment, the plasmid significantly enhanced U-2OS cell invasion, migration and proliferation (Figures 6A–D). All the results suggest that COLGALT2 may mediate the osteosarcoma-promoting effects of ADSC exosomes.

### ADSC Exosomes Trigger Osteosarcoma Progression by Activating COLGALT2 in Nude Mouse Models

**FIGURE 7:**
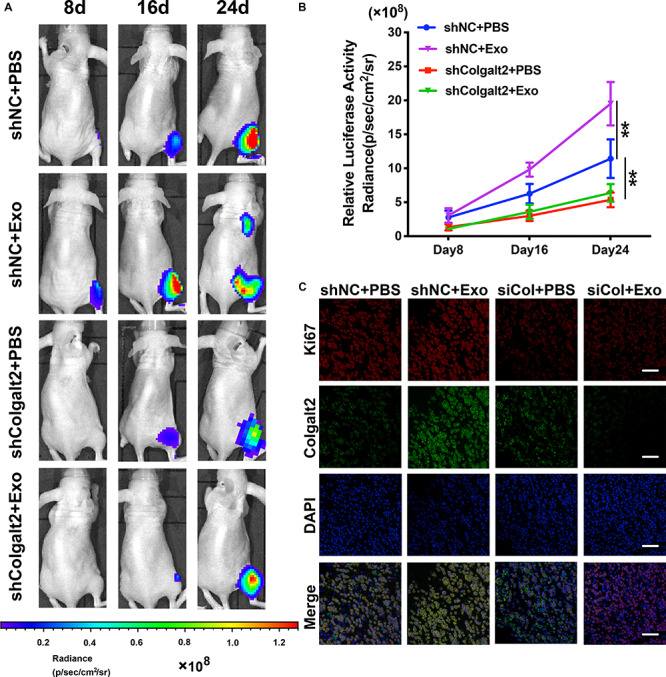
COLGALT2 mediates the promotion effects of ADSC exosomes on osteosarcoma metastasis and growth in models of nude mice. **(A)** To inspect osteosarcoma xenograft luminescence activity, an *in vivo* imaging system was applied. The results indicated tumor metastasis and growth. **(B)** Living Image Software was used to examine the tumor bioluminescence intensity every 8 days. Additionally, the quantitation of the normalized image counts is displayed. **(C)** The immunohistochemical analysis of COLGALT2 and Ki67 expression in orthotopic tumor xenografts is shown. Scale bar: 25 μm. **P < 0.01.

We constructed models of tibia osteosarcoma in nude mice and used human OS MG63 cells to determine how the osteosarcoma-promoting effects of ADSC exosomes are influenced by COLGALT2. Luciferase activity was examined with an IVIS imaging system to inspect the tumor cell volume. Compared with the control group, the signal grew more slowly in the shRNA-treated group and more quickly in the ADSC exosome-treated group (Figures 7A,B). Furthermore, more Ki67 was found in the tumor tissues of the ADSC exosome group, which was accompanied by higher COLGALT2 expression levels. These results suggested that these tumors grew faster. COLGALT2 inhibition significantly reduced the Ki67 and COLGALT2 levels (Figure 7C). Thus, ADSC exosomes promoted osteosarcoma metastasis and growth, and these effects were counteracted by COLGALT2 inhibition.

**FIGURE 8:**
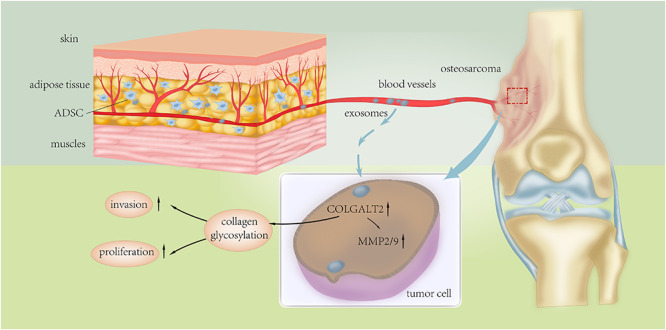
Schematic presentation of the mechanism underlying ADSC exosomes-facilitated osteosarcoma proliferation and metastasis.

## Discussion

Autologous adipose tissue transfer (ATT) may be performed for bone and joint reconstruction following osteosarcoma resection (Li and Nabet, 2019). In order to increase the efficiency of soft tissue defect correction, ATT is complemented with ADSCs (Liefhebber et al., 2010; Liu et al., 2019). Additionally, MSC-like cells can be applied in tumor-targeted cell therapy (Paino et al., 2017). However, the safety of autologous ATT in terms of the potential risk of cancer recurrence must be addressed, as ADSCs may have adverse effects on osteosarcoma development (Perrot et al., 2010; Liu et al., 2019). Several previous studies showed that conditioned medium from ADSCs increased the rate of osteosarcoma cell proliferation and invasion via soluble factors; however, the underlying mechanisms are still elusive (Schegg et al., 2008; Paino et al., 2017; Roma-Rodrigues et al., 2019). In this study, we focused on ADSC exosomes and demonstrated that they could induce COLGALT2 overexpression in osteosarcoma cell lines, which could increase osteosarcoma cell invasion, migration and proliferation *in vivo* and *in vitro*. In addition, higher expression of COLGALT2 was found to be associated with higher levels of vimentin and MMP2/9 in osteosarcoma cells. Although this finding still requires further investigation, we hypothesize that ADSC-secreted exosomes can induce COLGALT2 expression in osteosarcoma cells, accompanied by the downstream activation of vimentin and MMP2/9. The evidence shows that COLGALT2, which is overexpressed in metastatic osteosarcoma tissues, has great impacts on the tumor-promoting effects of ADSC exosomes.

Collagen proteins are the most abundant components of the extracellular matrix; therefore, the regulation of collagen modifications plays important roles throughout the body (Vettor et al., 2009). Because two beta (1-O) galactosyl-transferases were only recently discovered, research on the functions of COLGALT1 and COLGALT2 (also known as GLT25D1 and GLT25D2) is rare (Guo et al., 2019). As a beta (1-O) galactosyl-transferase that modifies collagen, COLGALT1 is reported to be involved in the development of mammary tumor metastases (Wang et al., 2017). Collagen glycosylation mediated by COLGALT2 is involved in the pathogenesis of inflammatory immune regulation and has been reported to regulate a subset of CD4 + T cells (Webster et al., 2017). Our results also confirmed that COLGALT2 may mediate the osteosarcoma progression induced by ADSC exosomes. Further studies should focus on the underlying mechanisms that mediate the immune regulation of COLGALT2 and facilitate cancer progression.

Our previous studies have reported that ADSCs in the osteosarcoma stroma could drive the growth and metastasis of tumors and that the soluble factors secreted by ADSCs may be mediated by changes in tumor cells (Collantes et al., 2018). However, it is still unclear whether the exosomes secreted by ADSCs are involved in the tumor-promoting effects of ADSCs. In this study, we confirmed that exosomes secreted by ADSCs had effects on osteosarcoma cells that were similar to those of ADSC-conditioned medium, and we found that exosomes mediated crosstalk between ADSCs and tumor cells. Furthermore, ADSC exosomes have been shown to induce the epithelial-mesenchymal transition and growth factors, such as vimentin and matrix metalloproteinases (MMPs), in tumor cells, which could partly explain the increased proliferation and invasion.

Altogether, our results support that exosomes secreted by ADSCs in adipose tissues can activate the expression of COLGALT2 in osteosarcoma cells, which fosters cancer metastasis and growth by increasing vimentin and MMP expression. Thus, our study demonstrates the function of collagen glycosylation in the regulation of the extracellular matrix by tumor cells and ADSCs in the tumor microenvironment during the development and progression of osteosarcoma. However, our knowledge regarding the exact mechanism of the actions of COLGALT2 that influence the progression and metastasis of osteosarcoma is limited. Our findings may lead to the design of therapies for osteosarcoma-targeting protein modifications to alter the tumor-promoting effects of ADSCs in the tumor microenvironment.

## Data Availability Statement

The raw data supporting the conclusions of this article will be made available by the authors, without undue reservation, to any qualified researcher.

## Ethics Statement

The study was approved by the Ethics Committee of the Qingdao University Affiliated Hospital. All participants provided written informed consent.

## Author Contributions

BC and YW designed the project. YC, HX, KL, and ZG developed the experiments. XW, CQ, YL, DZ, and GZ analyzed the data. YC and YW wrote the manuscript. XW and JS performed differentiations and analyzed the experiments.

## Conflict of Interest

The authors declare that the research was conducted in the absence of any commercial or financial relationships that could be construed as a potential conflict of interest.

## Supplementary Material

FIGURE S1 GAPDH mRNA levels in osteosarcoma cells treated with ADSCs exosomes or COLGALT2 shRNA were examined by qRT-PCR analysis.

FIGURE S2 COLGALT2 mRNA expression in ADSCs and MRC-5 cells exosomes were examined by qRT-PCR analysis.
