# Supplementary figures and images for: Exosomes Secreted by Adipose-Derived Mesenchymal Stem Cells Foster Metastasis and Osteosarcoma Proliferation by Increasing COLGALT2 Expression
Source: Front Cell Dev Biol. 2020 May 25;8:353. doi: 10.3389/fcell.2020.00353 (PMC7262406; doi:10.3389/fcell.2020.00353)

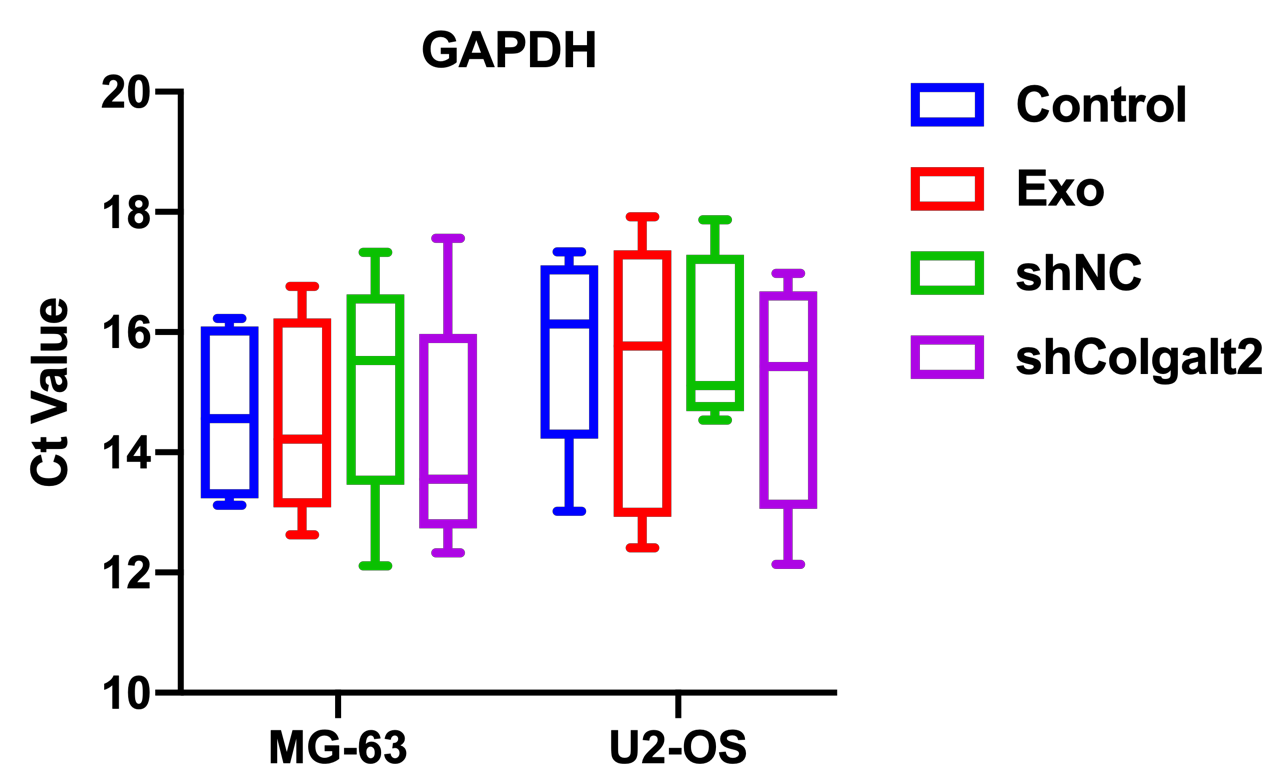

Supplement: FIGURE S1 — GAPDH mRNA levels in osteosarcoma cells treated with ADSCs exosomes or COLGALT2 shRNA were examined by qRT-PCR analysis. [file Image_1.TIFF]

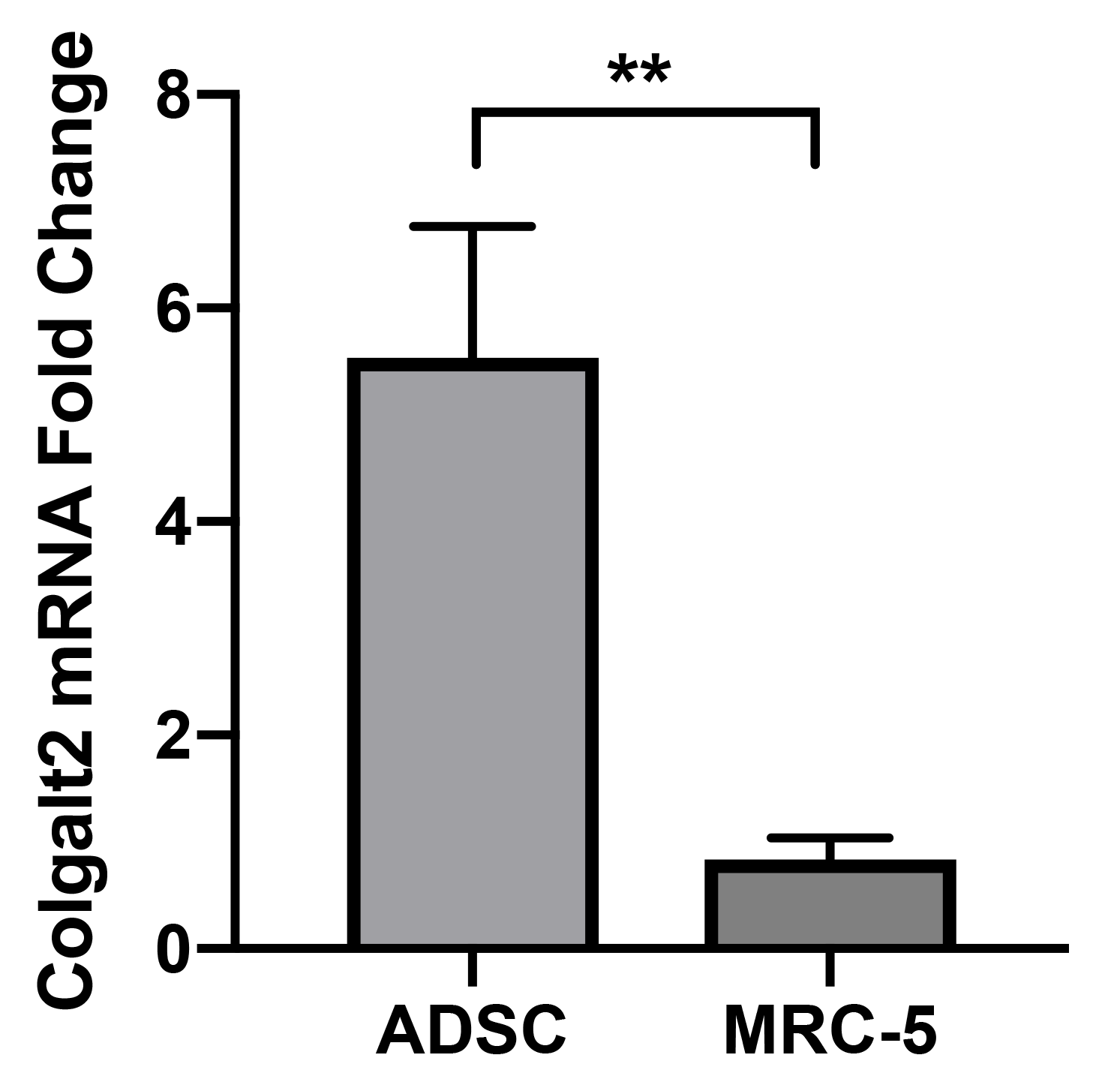

Supplement: FIGURE S2 — COLGALT2 mRNA expression in ADSCs and MRC-5 cells exosomes were examined by qRT-PCR analysis. [file Image_2.TIF]
