# Supplementary material for: Exosomes Secreted by Adipose-Derived Mesenchymal Stem Cells Foster Metastasis and Osteosarcoma Proliferation by Increasing COLGALT2 Expression
Source: Front Cell Dev Biol. 2020 May 25;8:353. doi: 10.3389/fcell.2020.00353 (PMC7262406; doi:10.3389/fcell.2020.00353)
Supplement: Supplementary file 3 [file Table_1.DOCX]

Table S1 Adipose Tissue Donor Information

| Dornor  (n) | Age  (Y) | Height  (cm) | Weight  (Kg) | BMI  (Kg/m^2^) | Gender (M/F) | Comments | Operation | Exosomes used |
| --- | --- | --- | --- | --- | --- | --- | --- | --- |
| 1 | 36 | 170 | 76 | 26.3 | M | Femoral shaft fracture | Internal Fixation of femoral shaft fracture | Yes |
| 2 | 34 | 155 | 70 | 30.1 | F | None | Liposuction operation | No |
| 3 | 20 | 166 | 62 | 22.4 | F | Femoral shaft fracture | Internal Fixation of femoral shaft fracture | Yes |
